# Electric-field-induced interferometric resonance of a one-dimensional spin-orbit-coupled electron

**DOI:** 10.1038/srep38851

**Published:** 2016-12-14

**Authors:** Jingtao Fan, Yuansen Chen, Gang Chen, Liantuan Xiao, Suotang Jia, Franco Nori

**Affiliations:** 1 State Key Laboratory of Quantum Optics and Quantum Optics Devices, Institute of Laser spectroscopy, Shanxi University, Taiyuan 030006, China; 2 Collaborative Innovation Center of Extreme Optics, Shanxi University, Taiyuan, Shanxi 030006, China; 3 State Key Laboratory of Quantum Optics and Quantum Optics Devices, Institute of Opt-Electronics, Shanxi University, Taiyuan, Shanxi 030006, China; 4 CEMS, RIKEN, Saitama 351-0198, Japan; 5 Department of Physics, University of Michigan, Ann Arbor, Michigan 48109-1040, USA

## Abstract

The efficient control of electron spins is of crucial importance for spintronics, quantum metrology, and quantum information processing. We theoretically formulate an electric mechanism to probe the electron spin dynamics, by focusing on a one-dimensional spin-orbit-coupled nanowire quantum dot. Owing to the existence of spin-orbit coupling and a pulsed electric field, different spin-orbit states are shown to interfere with each other, generating intriguing interference-resonant patterns. We also reveal that an in-plane magnetic field does not affect the interval of any neighboring resonant peaks, but contributes a weak shift of each peak, which is sensitive to the direction of the magnetic field. We find that this proposed external-field-controlled scheme should be regarded as a new type of quantum-dot-based interferometry. This interferometry has potential applications in precise measurements of relevant experimental parameters, such as the Rashba and Dresselhaus spin-orbit-coupling strengths, as well as the Landé factor.

Being an intrinsic property of condensed-matter materials, spin-orbit coupling (SOC) mixes the orbital and spin degrees of particles, and opens the possibility of electric control of the electron spin via its orbit, apart from the well-known magnetic responses^1^,^2^,^3^,^4^,^5^,^6^,^7^,^8^,^9^,^10^,^11^,^12^,^13^,^14^,^15^,^16^,^17^. A notable example exploiting SOC in semiconductor nanostructures is called the electric-dipole spin resonance technique^6^,^7^,^8^,^9^ (EDSR), in which a spin-orbit qubit is encoded into a SOC-hybridized spin doublet and an oscillating electric field is further applied to manipulate this qubit on its Bloch sphere. Recently, much theoretical^9^,^10^,^11^ and experimental^12^,^13^,^14^,^15^ attention have been paid to explore the EDSR in semiconductor quantum dot (QD). For example, utilizing this technique, the single spin-orbit qubit operation has been achieved^13^ and the spin-orbit effective field can also be determined^15^, which reflects its potential application in quantum information processing and parameters measurement. In addition, the SOC-assisted spin control, such as the magnetic-free spin filtering^16^,^17^ where the SOC serves as a necessary ingredient to spatially and electrically separate electrons with different spins, has also been achieved. In contrast to the conventional fully-magnetic control, the introduction of electric passage via SOC paves a much more experimentally feasible way to locally address electron spin, which may impact spintronics^18^.

Matter-wave interference exquisitely exhibits the wave nature of particles, which offers microscopic information of certain physical processes^19^,^20^,^21^,^22^,^23^. Various interferometries, for, e.g. electrons^24^,^25^,^26^,^27^,^28^,^29^,^30^,^31^,^32^, neutrons^33^,^34^, and atoms^35^,^36^,^37^, have been widely applied to measure various physical quantities, by virtue of their wave nature. With its rapid improvement of relevant experiment and theory, SOC, which exists naturally in condensed-matter systems^38^,^39^,^40^ and is also simulated in ultracold atomic systems^41^,^42^,^43^, is expected to be a new physical resource to demonstrate particle coherence in a spin-orbit-mixed way.

In this report, we theoretically formulate an electric mechanism to interfere electron orbits, by focusing on a one-dimensional (1D) spin-orbit-coupled nanowire QD. Owing to the existence of SOC and a pulsed electric field, different spin-orbit states are shown to interfere with each other, generating intriguing interference-resonant patterns. Furthermore, an in-plane magnetic field, treated as a perturbation, is also introduced to probe the relevant dynamics. We find that this magnetic field does not affect the interval of any neighboring resonant peaks, but contributes a weak shift of each peak, which is sensitive to the direction of the applied magnetic field. We find that this proposed external-field-controlled scheme, exhibiting all the basic ingredients of a quantum interferometer, should be regarded as multi-arm interferometry. We emphasize that the obtained interferometric signal originates from the out-of-phase interference of the dynamical phase factors of the infinite spin-orbit states, which is remarkably different from conventional optical/atomic interferometers^36^. This interferometry has potential applications in precise measurements of relevant experimental parameters, such as the Rashba and Dresselhaus SOC strengths, as well as the Landé factor.

## Results

The system we consider is a 1D nanowire QD with SOC, confined in a harmonic well and subjected to time-dependent external electric and magnetic fields. The total Hamiltonian can be divided into three parts^9^,^4^


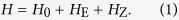


Here, the “free” Hamiltonian, without external fields, reads


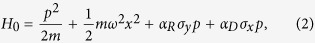


where *p* = −*iħ*∂/∂*x, m* is the effective electron mass, *α*_*R(D*)_ is the Rashba (Dresselhaus) SOC strength, and *σ*_*x(y*)_ is the Pauli spin operator. The Hamiltonian for the electric-dipole energy, induced by an external electric field 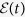, is written as


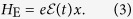


The Hamiltonian for the Zeeman energy of an electron, under an in-plane magnetic field **B**(*t*), is given by


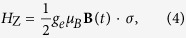


where *g*_*e*_ is the Landé factor, *μ*_*B*_ is the Bohr magneton, and **B**(*t*) = *B(t*)**n**, with **n** = (cos*θ*, sin*θ*, 0) being the direction of the external magnetic field.

It is convenient to introduce two auxiliary parameters,


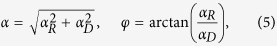


to rotate the spin space, along the *z* axis, to a new frame. In this case, the Hamiltonians (2) and (4) become


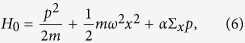


and


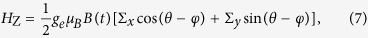


where ∑_*x*_ = *σ*_*x*_ cos*φ* + *σ*_*y*_ sin*φ* and ∑_*y*_ = −*σ*_*x*_ sin*φ* + *σ*_*y*_ cos*φ* are the redefined spin operators in the new frame, while the Hamiltonian (3) remains unchanged. Since SOC endows the quantum dot with the ability to respond to both the external electric and magnetic fields, our goal here is to build a new-type of quantum-dot-based interferometer by utilizing this natural response.

Before specifying the temporal shapes of the external fields, we first analyze the “unpertubed” Hamiltonian *H*_0_, under which the initial state is prepared. Taking into account the conservation of the redefined spin operator Σ_*x*_, the eigenstates of the Hamiltonian *H*_0_ are represented as


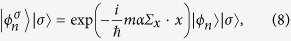


where the orbital part |*ϕ*_*n*_〉 is the *n*th eigenstate of a harmonic oscillator and the spin part |*σ*〉 is the eigenstate of the redefined spin operator Σ_*x*_, i.e., ∑_*x*_|*σ*〉 = *σ*|*σ*〉, with *σ* = ±1. Notice that for each electron orbit, the total eigenstates are twofold degenerate. We assume that the “unpertubed” system is initially prepared in its ground state (*n* = 0), with a general superposition of two spin components, i.e., 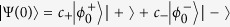, where |*c*_+_|^2^ + |*c*_−_|^2^ = 1.

To run the dynamics, we turn-on the external fields at a certain time *t*_0_. In our proposed interferometer, the external fields are utilized as “phase objects” to generate proper interferometric phases^36^, and their detailed field profiles should be well engineered. As an instructive example, the electric and magnetic signals are now taken as


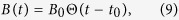



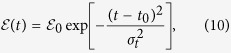


where Θ(*t* − *t*_0_) is the Heaviside step function, and 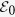 and *σ*_*t*_ are the peak amplitude and temporal width of the Gaussian-type pulse, respectively. The above expressions of the external fields show that the magnetic field, characterized by the constant field strength *B*_0_, looks like a simple quantum “quenching knob”. whose effect is quite different from the electric field. This different choice of the electric and magnetic fields relies on their individual roles in activating novel dynamics in the nanowire QD, as will be described below.

To further facilitate the theoretical description, we prefer to transform the Hamiltonian (1) to the frame of the “velocity” gauge by using a unitary operator 
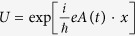
, with the gauge potential


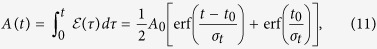


where 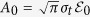 and erf(*x*) is the error function. After performing the transformation 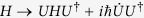, the Hamiltonian (1) becomes


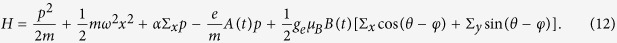


Despite of its simple form, the Hamiltonian (12) still governs a quite complicated dynamics; so much so that no exact solution can be found. To catch the basic idea of the proposed interferometric process, we simplify the analysis over two aspects. Firstly, we restrict the Zeeman energy, 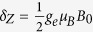, to a weak regime such that it is much less than the orbital splitting, i.e., 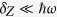. Therefore, the Hamiltonian contributed by the magnetic field can be treated perturbatively. Secondly, from the expression of *A(t*), we find that in the limit *σ*_*t*_ → 0 and 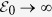 (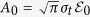 still remains finite), the gauge potential tends to be *A(t*) = *A*_0_ Θ (*t* − *t*_0_), and the corresponding electric field becomes a delta-type pulse, i.e., 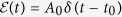. It follows that under such a condition, both the electric and magnetic manipulations become external quantum quenching knobs, and furthermore, this ultrafast limit of the electric-field pulse allows us to obtain a compact analytical solution, which captures the key aspects of this interferometer.

Since the control parameters *A(t*) and *B(t*) are switched on at time *t*_0_ and thereafter remain constant, the total dynamics can be conveniently simplified to two stationary problems of times *t* < *t*_0_ and *t* > *t*_0_, which are governed respectively by the Hamiltonians *H(t* < *t*_0_) and *H(t* > *t*_0_). We now employ perturbation theory to solve these. We first concentrate on the zeroth-order Hamiltonian *H*_S_ = *H*_0_ + *H*_E_. Notice that without the perturbed magnetic field, the redefined spin operator Σ_*x*_ commutes with the Hamiltonian *H*_S_. In terms of this, the eigenstates of the Hamiltonian *H*_S_(*t* > *t*_0_) are obtained exactly by


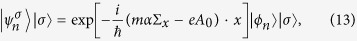


with eigenenergies


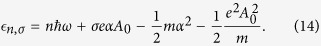


Obviously, the electric field lifts the degeneracy of each orbital energy by 2*eαA*_0_. However, there exists a special case where the *n*th and (*n* + *k*)th orbits are degenerate or quasi-degenerate, say 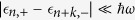 (see the Methods Section). In such case, the non-degenerate perturbative formula breaks down and we must use a degenerate perturbation method. Therefore, the complete solutions should be divided into a nondegenerate case (NC) and a degenerate/quasi-degenerate case (DC). After a straightforward calculation, the eigenstates of the Hamiltonian *H(t* > *t*_0_), which are accurate up to first order in the Zeeman energy *δ*_*Z*_, can be summarized as


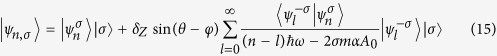


for the NC and


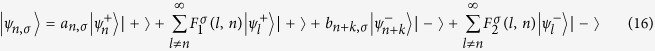


for the DC, where *a*_*n,σ*_, *b*_*n,σ*_, and 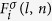 (*i* = 1, 2) are given in the Methods Section. Furthermore, the corresponding perturbative eigenenergies are given by (see also the Methods Section for details)


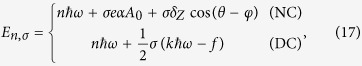


where


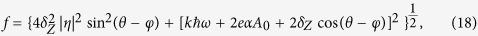


with 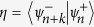. Notice that, for simplicity, here we have neglected the dependence of *f* on *n*, since *η* is very small for any *n* and *k*. Equation (17) shows that the external electric and magnetic fields dominate the dynamics through different ways: the former (electric) appears as a weight factor of the SOC strength, whereas the effect of the magnetic field depends crucially on its specific direction. These features clearly signal their quite different roles in controlling the interference pattern.

**Figure 1:**
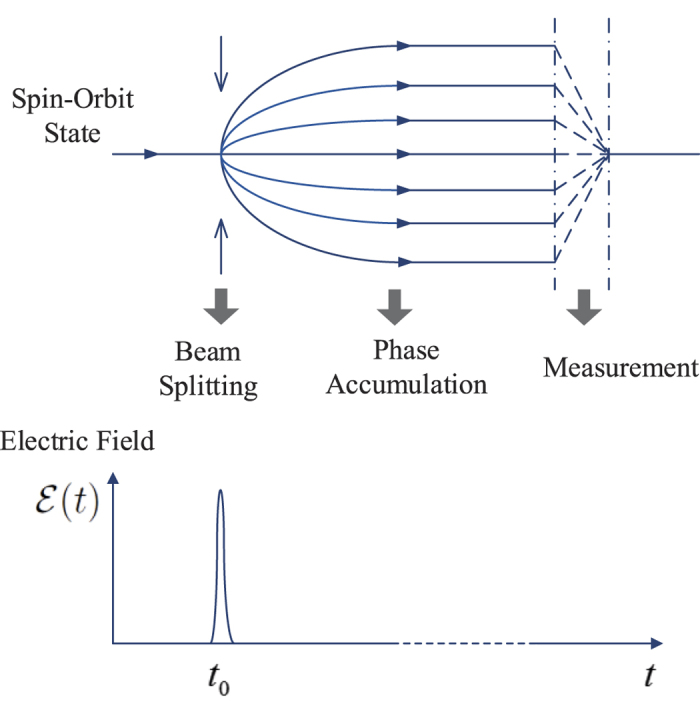
Schematic description of the proposed interferometer. Top panel: The initial spin-orbit state, staying in the zeroth orbit, is split into different orbital states at time *t*_0_. Each orbital state evolves independently in its individual passage, accumulating a relative phase shift. The interference pattern arises from a specific measurement, which also acts as a beam recombiner. Bottom panel: Timing of the pulsed electric field, which shifts the original orbit and plays the role of a beam splitter.

Having obtained the complete eigenstates and eigenenergies of the quenched Hamiltonian *H(t* > *t*_0_), we are now able to discuss the total dynamics of the system. After the quantum quench, the whole information of the nanowire QD with SOC is encoded in its instantaneous wavefunction, which can be expanded using the spin-orbit basis |*ψ*_*n,σ*_〉, i.e.,


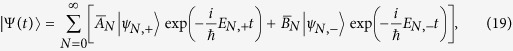


where 
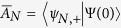
 and 
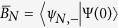
 are the projected coefficients for the “+” and “−” spin sectors of the *N*th orbit, respectively. A crucial point we should notice is that being a direct consequence of the pulsed electric field, 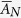 and 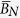 may acquire non-zero values even for *N* ≠ 0. In other words, it is the pulsed electric field at time *t*_0_ that splits the original zeroth-orbit wavefunction into other different orbital states |*ψ*_*n,σ*_〉. Indeed, in the nanowire QD with SOC, we have built a *multiple-polarization-interferometer*, where the interferometric arms correspond to infinite different orbital states, and the beam splitter corresponds to the pulsed electric field (see Fig. 1 for a more intuitive description). However, to obtain a signature of interference, usually observed as a population difference of a physical quantity, a special operation to recombine the split orbital states is still needed. Motivated by the fact that any different spin-reversed orbital states are non-orthogonal (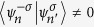) due to the existence of SOC, it is thus convenient to investigate the ensemble average of the spin polarization *σ*_*z*_. The result is given by


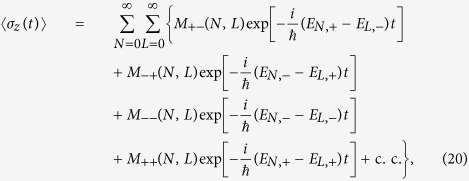


where *M*_*αβ*_(*N, L*) (*α, β* = ±) are given in the Methods Section and c.c. denotes the complex conjugate.

As shown in Eq. (20), 〈*σ*_*z*_(*t*)〉 exhibits the inner product between different spin-reversed orbital states, namely 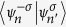, which is of great importance to support the accumulated dynamical phase factors. Evidently, these phase factors will lead to periodic oscillations of 〈*σ*_*z*_(*t*)〉 over time, which may be referred to as (time-domain) interference fringes. However, instead of focusing on the time-dependent interference signals, usually done in time-domain Ramsey-like atom interferometry^44^,^45^, we prefer to explore the long-time average of 〈*σ*_*z*_(*t*)〉,


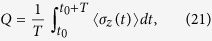


where *T* is a long timespan, to extract a more prominent interference-resonant effect.

**Figure 2:**
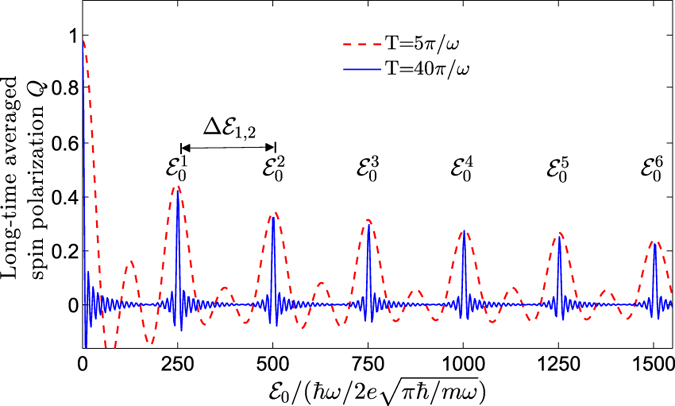
The long-time averaged spin polarization *Q* versus the peak amplitude 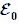. Here, the SOC strength, the temporal width, and the initial state are given by 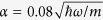, *σ*_*t*_ = 0.05/*ω*, and 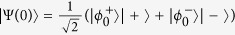, respectively. The timespans for the red-dashed and blue-solid curves are chosen as *T* = 5*π/ω* and 40*π/ω*, respectively.

Figure 2 shows the evolution of *Q* versus 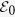, by numerical integration of the time-dependent Schrödinger equation, with 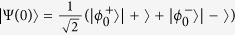 and *T* = 40*π/ω* (blue-solid curve). It is remarkable to see that the interference pattern appears, and more interestingly, some sharp interference-resonant peaks are formed periodically at


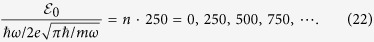


The underlying physics of such resonant effect should be traced back to the out-of-phase interference, contributed by the continuous dynamical phase factors. To see this clearly, we first note that in Eq. (20) there exists two kinds of phase factors, 
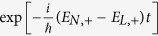
 and 
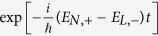
, of which the latter involves essential information of the external fields, whereas the former does not. In fact, for general values of 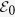 and *B*_0_, the oscillating frequencies of the latter, (*E*_*N*,+_ − *E*_*L*,−_)/*ħ*, are nonzero and usually significantly large. Thus, both the first and second terms in Eq. (20) vanish after long-time averaging, due to the out-of-phase interference. However, when 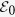 is tuned to some specific values, such that


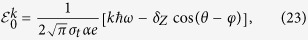


with *k* = *L* − *N*, the system reaches its level (avoided) crossing point, i.e., *E*_*N*,+_ − *E*_*L*_ = 2*δ*_*Z*_|*η*|sin(*θ* −*φ*) ≈ 0, which can be calculated from the second line of Eq. (17). At this point, the out-of-phase interference is maximally suppressed [the out-of-phase part vanishes if *E*_*N*,+_ − *E*_*L*,−_ = 0], giving rise to a considerable non-zero contribution to *Q*, and the interference-resonant peaks thus emerge.

**Figure 3:**
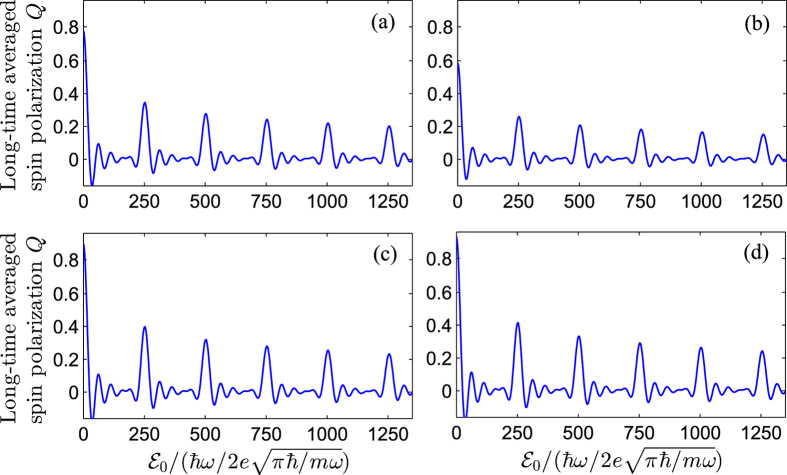
The long-time averaged spin polarization *Q* versus the peak amplitude 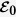 for four different initial states. In these plots, *c*_+_/*c*_−_ is given by (**a**) 2/1, (**b**) 3/1, (**c**) 2/3, and (**d**) 3/4, respectively. The timespan is chosen as *T* = 10*π/ω*. Other parameters are the same as those in Fig. 2.

The tunability of the widths of the resonant peaks, which favors its experimental observability, can be achieved by varying *T*. As shown in Fig. 2 by the red-dashed curve, a shorter averaging timespan, *T* = 5*π/ω*, results in broader resonant peaks but does not change their peak positions. We emphasize that the above analysis is general and independent of the specific values of 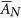 and 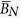. That is to say, different choices of the values of *c*_+_ and *c*_−_ of the initial state, except for a special case *c*_+_ · *c*_−_ = 0, only lead to different amplitudes of the resonant peaks rather than their specific positions, which has also been confirmed by direct numerical simulations (see Fig. 3). Our main results, therefore, are robust and will not be affected.

**Figure 4:**
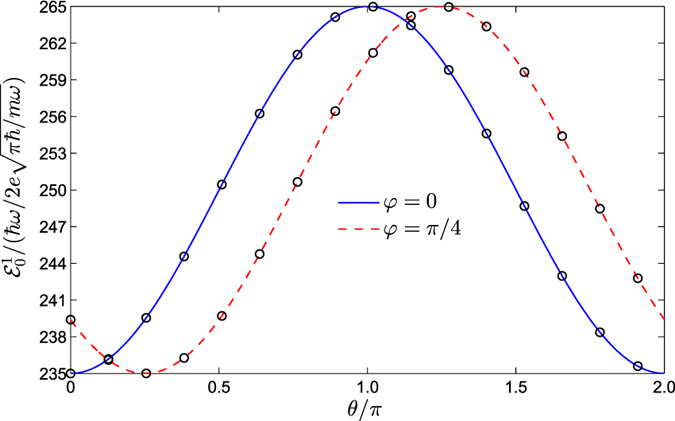
The position of the second resonant-peak 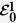 versus the magnetic-field direction *θ*. Here, the timespan and the timespan to turn on the magnetic field are given by *T* = 10*π/ω* and *τ* = 4.3/*ω*, respectively. The SOC strength *α*, the temporal width *σ*_*t*_, and the initial state |Ψ(0)〉 are the same as those in Fig. 2. The black open circles correspond to direct numerical simulations, while the curves show our analytical results in Eq. (23).

From Eq. (23), we find that the interval of any neighboring resonant peaks,


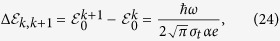


remains a constant irrespective of the magnetic field, but depends crucially on the SOC. However, the position of each individual resonant peak is shifted by a small value via the Zeeman interaction, which is sensitive to the direction of the magnetic field. The above two features of the proposed interferometer provide a meaningful method to precisely measure relevant experimental parameters. A typical example is the determination of the Rashba and Dresselhaus SOC strengths, which is of critical importance in current condensed-matter experiments^46^,^47^,^48^,^49^. To this end, we first note that the total SOC strength is obtained explicitly through 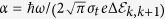 by measuring 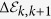. Moreover, using the relation between the resonant-peak position and the magnetic-field direction, we can further determine the strengths of the Rashba and Dresselhaus SOCs. In Fig. 4, we numerically monitor the response of the position of the second resonant-peak 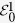 on the magnetic-field direction *θ*, by using *δ*_*Z*_ = 0.06*ħω*. Bearing in mind the existence of a finite time to turn on the magnetic field in a realistic situation, in our numerical simulation we have intentionally replaced the Heaviside temporal shape of *B(t*) by an exponential-ramped timing, namely,


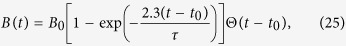


with *τ* being the timespan to turn on the magnetic field [the factor 2.3 in Eq. (25) ensures that *B(t*_0_ + *τ*)/*B*_0_ = 0.9]. Obviously, in the limit *τ* → 0, we recover the condition *B(t*) = *B*_0_ Θ (*t* − *t*_0_). As shown in Fig. 4, for *τ* = 4.3/*ω*, the direct numerical simulations agree well with the analytical expression in Eq. (23), implying that the Heaviside function Θ(*t* − *t*_0_) is a good approximation of a realistic situation. Being similar to the previous results^9^, the position of the resonant peak reaches its extreme value at *θ*_*m*_ = *nπ* + *φ (n* = 0, ±1, 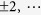), and *φ* is thus determined from the obtained *θ*_*m*_. Having obtained the auxiliary parameters *α* and *φ*, the Rashba and Dresselhaus SOC strengths are expressed directly as *α*_*R*_ = tan*φ* and *α*_*D*_ = cot*φ*, respectively. Obviously, a similar method can also be employed to determine the Landé factor, since Eq. (23) also contains this basic information.

Note that in the case of *θ* = *θ*_*m*_, the Hamiltonian (12) is exactly solvable due to the cancelation of the redefined spin operator Σ_*y*_. Further calculations accordingly prove the validity of Eqs (17) and (23), without any limitation on *B*_0_. In this case, using Eq. (23), it seems that similar magnetic-field-driven resonant peaks would arise at 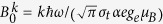 without introducing the pulsed electric field. However, this straightforward derivation is not valid. Unlike the pulsed electric field, which serves as a beam splitter in the proposed interferometer, the magnetic field does not shift the original orbit (see the expression of |*ψ*_*n,σ*_〉), and thus 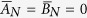 for any *N* ≠ 0 in Eq. (20). This implies that all the dynamical phase factors in Eq. (20) cancel out, and the interference-resonant peaks vanish. Therefore, it can be seen that the pulsed electric field plays a unique role in inducing transitions between the external orbital states, which is the key to switching on the interferometric process.

Based on current experimental conditions of nanowire QDs, we now estimate various relevant parameters to show the experimental feasibility of our proposal. Consider the material parameters of GaAs^50^, namely, *g*_*e*_ = −0.44, *α* = 1.83 × 10^−11^ eV · cm/*ħ, m* = 0.067*m*_0_, where *m*_0_ is the electron mass, and assume a weak trap potential *ħω* = 9.1 *μ*eV, which can be controlled by gate voltages^13^. Therefore, the width of the electric-field pulse and the timespan to turn on the magnetic field is estimated respectively as *σ*_*t*_ = 0.05/*ω* ≈ 4 ps and *τ* = 4.3/*ω* ≈ 300 ps, which is experimentally reasonable in view of the fact that ultrafast field pulses about the order of picosecond have been reported^51^. Accordingly, a viable averaging timespan can be chosen as *T* = 10*π/ω* = 2.2 ns, which is shorter than the spin dephasing time 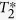 in GaAs QD, which typically is ~10 ns^52^. This confirms the observation of the predicted interferometric resonance within the spin coherence time. In terms of the above parameters, the interval of the resonant peaks plotted in Fig. 2 is also given by 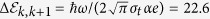 V/cm. Since the Landé factor of GaAs is very small (*g*_*e*_ = −0.44), a weak Zeeman energy, *δ*_*Z*_ = 0.06*ħω*, still supports a considerably-strong magnetic field *B* = 42 mT, which in turn shifts the resonant peaks by, as large as, 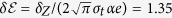 V/cm. Moreover, considering the fact that the electric field in current experiments can easily reach ~10^5^ V/cm, our interferometric method can thus be applied to precisely measure materials with even much weaker SOC strengths. The relevant parameters of some other semiconductor materials, namely GaAs, InSb, InAs, ZnO, and GaN, can be found in Table 1.

## Discussion

**Figure 5:**
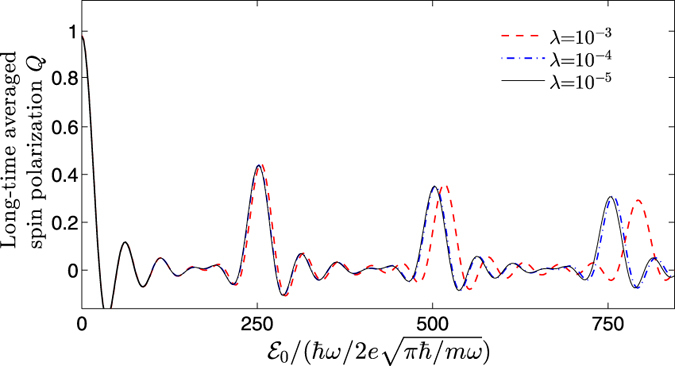
The long-time averaged spin polarization *Q* versus the peak amplitude 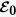 with respect to different anharmonic factors, *λ* = 10^−3^ (red dashed curve), *λ* = 10^−4^ (blue dotted-dashed curve) and *λ* = 10^−5^ (black solid curve). The timespan is chosen as *T* = 10*π/ω*. Other parameters are the same as those in Fig. 2.

Taking into account the fact that the anharmonicity is unavoidable in an actual experiment, we add a higher order anharmonic factor, *βx*^4^, in the Hamiltonian (12), i.e.,


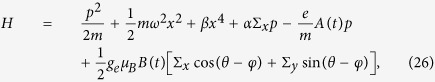


to numerically analyze its impact on the interference peaks. To be clarity, a dimensionless parameter, *λ* = 2*βħ*/(*m*^2^*ω*^3^), is introduced. Figure 5 shows the interference patterns with respect to different *λ*. It can be seen that (i) the first few peaks are stable if *λ* is relatively small and with the increasing of *λ*, the peaks tend to deviate from their standard values of the harmonic case; (ii) the higher resonant peaks are more sensitive to the anharmonic perturbation than the lower ones. These behaviors can be qualitatively explained as follows. The effect of the anharmonicity can be neglected only if the quartic term 〈*βx*^4^〉 is effectively small enough than 〈*mω*^2^*x*^2^/2〉 (〈…〉 means expectation value). With the increasing of 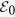, the wave function is more likely to be excited to the higher orbit states (see the expressions of 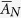 and 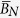) and therefore becomes more extended, which consequently highlights the impact of the quartic term 〈*βx*^4^〉. On the other hand, when the orders of *λ* is no more than 10^−3^, the first two resonant peaks are definitely stable, reflecting their robustness under the anharmonicity. As we elucidated in the previous section, the knowledge of the first two peaks is sufficient to extract information of the considered SOC electron.

Note that various techniques have been employed to determine the Rashba and Dresselhaus SOC strengths^13^,^46^,^47^,^48^,^49^. We emphasize here, however, that our proposal is much different from previous schemes in principle. Firstly, unlike previous works, in which the spin precession responding to external magnetic fields is mainly investigated^47^,^48^,^49^, the physics we exploited in this report essentially originates from the interference of the dynamical phase factors of different orbital states. The electric field here serves as a basic building block, say, the beam splitter, of a multiple-polarization-interferometer. Secondly, the existing schemes mostly extract information from the instantaneous spin evolution, while the novel spin dynamics prediceted in this work reflects in its long-time mean value.

Finally, we emphasize that the proposed idea of the SOC-induced multiple-polarization quantum interferometer is general and the 1D nanowire QD just offers a platform to demonstrate the relevant physics. Actually, the model Hamiltonian (1) should, by no means, be limited to only a single specific system. Resent advances in ultracold atoms, with artificial gauge fields, make it another alternative candidate to exhibit the same physics. For example, the harmonic-trapped two-component Bose-Einstein Condensation (BEC), with synthetic 1D SOC, can be well simulated by the free Hamiltonian (6)^42^,^43^, and furthermore, a rapid shake of the harmonic trapping potential of the BEC ideally corresponds to the pulsed electric field 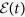 employed in the nanowire QD. Thus, following similar procedures we discussed above, a BEC-based interferometric resonance, with respect to the strength of the shake, can also be expected.

In summary, we have theoretically formulated an electron-orbital interferometry, by focusing on a 1D nanowire QD with SOC. By properly adjusting the external fields’ timing, different spin-orbit states are shown to interfere with each other, generating intriguing interference-resonant patterns. We have also shown that this interferometry has potential applications in precise measuring relevant experimental parameters, such as the Rashba and Dresselhaus SOC strengths, as well as the Landé factor.

## Methods

### Derivation of the perturbed eigenstates in the degenerate/quasi-degenerate case

**Figure 6:**
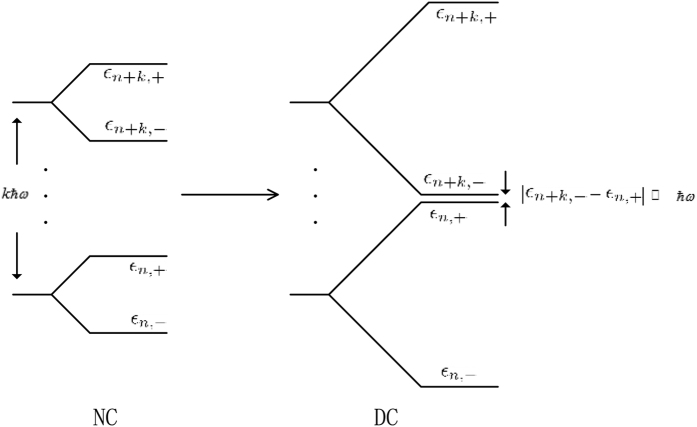
Schematic energy levels of the *n*th and (*n* + *k*)th orbits in the nondegenerate case (NC) and degenerate/quasi-degenerate case (DC).

By varying the parameters in Eq. (14), it is possible to achieve a regime where the *n*th and (*n* + *k*)th orbits are degenerate/quasi-degenerate, say 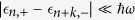; see Fig. 6. In such case, we must recombine the unperturbed eigenstates 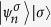 to obtain proper zeroth-order eigenstates.

We assume that the *n*th perturbed eigenstate can be expressed as


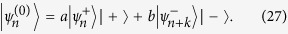


Substituting the assumed eigenstate in Eq. (27) into the Schrödinger equation 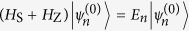 and making use of 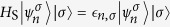, we obtain the following two equations:


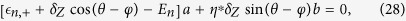



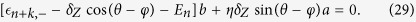


The appearance of nonzero solutions in Eqs (28) and (29) requires


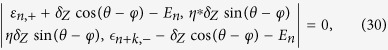


which results in


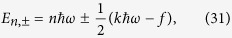


where


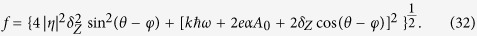


The corresponding eigenstates are given by


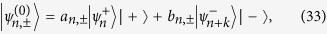


where


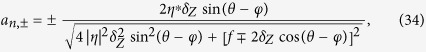



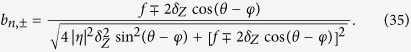


Based on the rearranged zeroth-order eigenstates 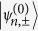 in Eq. (33), the first-order eigenstates are derived straightforwardly from perturbation theory. The results are given by


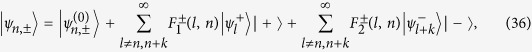


where


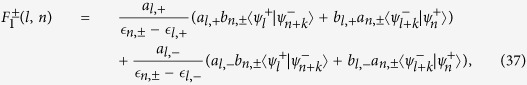


and


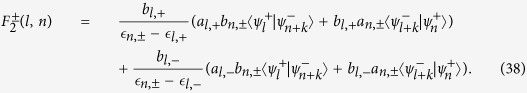


### Complete expression of Eq. (20) in the Results Section

The detailed expressions of *M*_*αβ*_(*N, L*) (*α, β* = ±) in Eq. (20) should be divided into the following two cases: (i) non-degenerate case, where each orbital energy 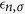 is well separated from others, and (ii) degenerate/quasi-degenerate case, where 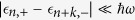. Specially, in the non-degenerate case we have


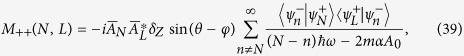



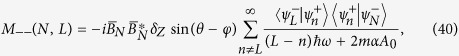



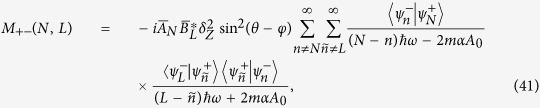



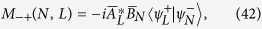


and in the degenerate/quasi-degenerate case we obtain


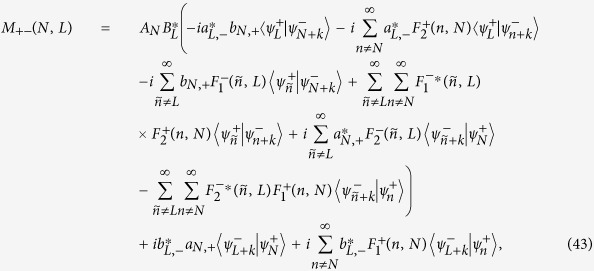



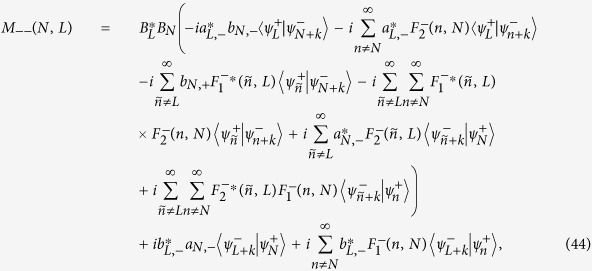



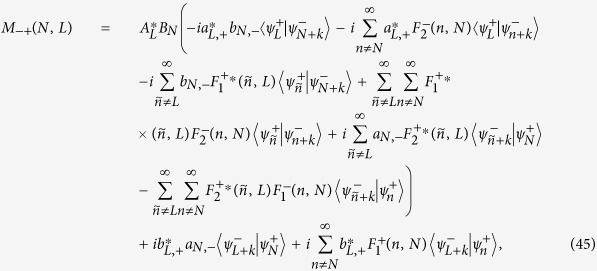



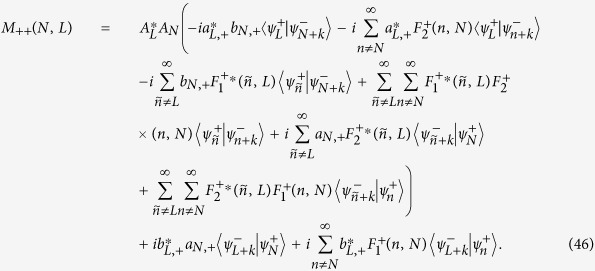


## Additional Information

**How to cite this article**: Fan, J. *et al*. Electric-field-induced interferometric resonance of a one-dimensional spin-orbit-coupled electron. *Sci. Rep.* **6**, 38851; doi: 10.1038/srep38851 (2016).

**Publisher's note:** Springer Nature remains neutral with regard to jurisdictional claims in published maps and institutional affiliations.

## Figures and Tables

**Table 1:** Some quantum dot parameters.

Semiconductor	*ħα*_*R*_ (eV · cm)	*ħα*_*D*_ (eV · cm)	*γ* (eV · Å ^3^)	*ħα* (eV · cm)	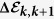 (V/cm)
GaAs	0.68 × 10^−11^^a^	−1.7 × 10^−11^	−11^a^	1.83 × 10^−11^	22.6
InSb	3 × 10^−10^^b^	7.7 × 10^−10^	490^b^	8.3 × 10^−10^	0.5
InAs	5.71 × 10^−9^^c^	9.0 × 10^−10^	571.8^c^	5.78 × 10^−9^	0.07
ZnO	1.1 × 10^−11^^d^	5.2 × 10^−13^	0.33^e^	1.1 × 10^−11^	37.6
CaN	9.0 × 10^−11^^f^	5.0 × 10^−13^	0.32^e^	9.0 × 10^−11^	4.6

The Dresselhaus SOC strength is estimated by *ħα*_*D*_ ≈ *γ(π/z*_0_)^2^, where *γ* is the material-specific constant and *z*_0_ is the quantum well vertical width. In these estimations, we assume *z*_0_ = 25 nm, *ħω* = 9.1 *μ*eV and *σ*_*t*_ = 4 Ps.

^a^ref. 50.

^b^ref. 53.

^c^ref. 54.

^d^ref. 55.

^e^ref. 56.

^f^ref. 57.
